# Cognitive and Motor Cortical Activity During Cognitively Demanding Stepping Tasks in Older People at Low and High Risk of Falling

**DOI:** 10.3389/fmed.2021.554231

**Published:** 2021-07-12

**Authors:** Paulo H. S. Pelicioni, Stephen R. Lord, Daina L. Sturnieks, Bethany Halmy, Jasmine C. Menant

**Affiliations:** ^1^Neuroscience Research Australia, University of New South Wales, Sydney, NSW, Australia; ^2^School of Population Health, University of New South Wales, Sydney, NSW, Australia; ^3^School of Physiotherapy, Division of Health Sciences, University of Otago, Dunedin, New Zealand; ^4^School of Medical Sciences, University of New South Wales, Sydney, NSW, Australia

**Keywords:** functional near infrared spectroscopy, aged, frailty, accidental falls, stepping, dorsolateral prefrontal cortex

## Abstract

**Background:** Choice stepping reaction time tasks are underpinned by neuropsychological, sensorimotor, and balance systems and therefore offer good indices of fall risk and physical and cognitive frailty. However, little is known of the neural mechanisms for impaired stepping and associated fall risk in older people. We investigated cognitive and motor cortical activity during cognitively demanding stepping reaction time tasks using functional near-infrared spectroscopy (fNIRS) in older people at low and high fall risk.

**Methods:** Ninety-five older adults [mean (SD) 71.4 (4.9) years, 23 men] were categorized as low or high fall risk [based on 12-month fall history (≥2 falls) and/or Physiological Profile Assessment fall risk score ≥1]. Participants performed a choice stepping reaction time test and a more cognitively demanding Stroop stepping task on a computerized step mat. Cortical activity in cognitive [dorsolateral prefrontal cortex (DLPFC)] and motor (supplementary motor area and premotor cortex) regions was recorded using fNIRS. Stepping performance and cortical activity were contrasted between the groups and between the choice and Stroop stepping conditions.

**Results:** Compared with the low fall risk group (*n* = 71), the high fall risk group (*n* = 24) exhibited significantly greater DLPFC activity and increased intra-individual variability in stepping response time during the Stroop stepping task. The high fall risk group DLPFC activity was greater during the performance of Stroop stepping task in comparison with choice stepping reaction time. Regardless of group, the Stroop stepping task elicited increased cortical activity in the supplementary motor area and premotor cortex together with increased mean and intra-individual variability of stepping response times.

**Conclusions:** Older people at high fall risk exhibited increased DLPFC activity and stepping response time variability when completing a cognitively demanding stepping test compared with those at low fall risk and to a simpler choice-stepping reaction time test. This increased hemodynamic response might comprise a compensatory process for postural control deficits and/or reflect a degree of DLPFC neural inefficiency in people with increased fall risk.

## Introduction

Frailty is a state of vulnerability to poor resolution of homoeostasis after a stressor event and is a consequence of cumulative decline in many physiological systems during a lifetime (1). Frail older people have high levels of disability, reduced ability to perform activities of daily living, restricted participation in life roles, and increased rates of hospitalization and institutionalization (2–4). Falls are also a major consequence of frailty (1), and it has been reported that falls and frailty have many shared risk factors including muscle weakness, instability, and impaired cognition (5).

The ability to generate quick and accurate steps to negotiate environmental hazards is particularly impaired in older people at high fall risk. For example, in a choice stepping reaction time (CSRT) task requiring participants to step as quickly as possible in response to visual targets, older people at high risk of falls were slower to step and made more stepping errors when their attention was divided compared to those at low risk of falls (6). Furthermore, poor performance in a CSRT task involving a Stroop condition that required stepping response inhibition has also been found to discriminate between fallers and non-fallers (7).

Systematic review evidence indicates that white matter lesions are significantly associated with impaired balance, gait, and mobility in older people with lesions in the frontal lobe and periventricular regions showing the strongest relationships with such motor impairments (8). Some studies have also reported that greater white matter lesion burden and/or sub-cortical infarcts predict falls over 12 months (9–11). Functional near-infrared spectroscopy (fNIRS)—a portable optical neuroimaging technique that enables investigation of cortical activity while participants move freely—is a valuable tool for assessing central nervous system factors that increase fall risk (12–14). However, only one study has investigated whether cortical activity using fNIRS during a walking condition is associated with falls in older people. This study found elevated prefrontal cortex (PFC) activity while walking and talking predicted falls over a 4-year period (15).

To date, very few studies have been conducted using fNIRS with stepping tasks (16–18). Yet, CSRT tasks may constitute appropriate models for investigating cortical activation patterns in a frailty context as they are composite measures of fall risk and are underpinned by neuropsychological, sensorimotor, and balance systems (19). As voluntary stepping is less automated than walking, it likely relies on an indirect locomotor pathway (14), comprising the dorsolateral prefrontal cortex (DLPFC) [involved in executive functioning including inhibitory processes (20)], and other cortical areas, such as the supplementary motor area (SMA) [involved in planning and generation of internally driven actions, including anticipatory postural adjustments at gait initiation, cf. SMA (21–23)] and the premotor cortex (PMC) [involved in sequencing of movements activated by external stimuli (24)]. It has previously been reported that a complex CSRT task compared to a simpler stepping reaction time task led to increased cortical activity in the DLPFC (17, 18), the SMA (18), and the PMC (18) in young (17) and older people (18). However, whether increased physical frailty results in compensatory increased reliance on these cortical areas during choice-stepping tasks that are composite measures of fall risk is unknown.

Therefore, the aims of this study were to compare cortical activity in the DLPFC, SMA, and PMC using fNIRS in older people at low and high fall risk during two stepping reaction time tasks that differed with respect to their cognitive challenge. In line with our previous work (6, 7), we hypothesized that compared to older people at low fall risk, older people at high fall risk would have slower and more variable step responses when undertaking both the simpler (CSRT) and more complex [Stroop stepping task (SST)] stepping tasks but that the between-group differences would be greater for the more complex stepping test. In addition, consistent with the Compensation-Related Utilization of Neural Circuits Hypothesis (CRUNCH) (25, 26), we hypothesized that older people at high fall risk would exhibit greater activity in all three cortical areas when undertaking both CSRT and SST stepping tasks and again the between-group differences would be greater for the SST. This pattern of neural and stepping responses would indicate that older people at high fall risk need greater cortical input to perform cognitively demanding stepping tasks [ “compensatory over-activation” (25)] but, despite such compensation, cannot perform the stepping tasks as well as older people at low fall risk [ “neural inefficiency” (25)].

## Materials and Methods

### Participants

The sample comprised 95 healthy older people [mean age (SD) = 71.3 (4.9) years, 23 men] who were living in Sydney, Australia, and recruited to participate in a randomized controlled trial of cognitive-motor interventions to prevent falls (ACTRN12616001325493) (27). Inclusion criteria were as follows: aged 65 years or over, living independently in the community, and able to communicate in English. Exclusion criteria were as follows: progressive neurological disorders, unstable medical or psychiatric conditions, and a Pfeiffer Short Portable Mental Status Questionnaire score <8 (28). The University of New South Wales Human Research Ethics Committee approved this study, and all participants gave informed consent prior to study participation.

### Assessment of Fall Risk

Fall risk was assessed using the Physiological Profile Assessment (PPA), which comprises five validated tests: visual contrast sensitivity, lower limb proprioception, knee extension strength, hand simple reaction time, and postural sway when standing on a compliant surface with eyes open for 30 s. A combined weighted score of these five measures provides an estimate of physiological fall risk and has been shown to have 75% accuracy in predicting multiple falls in older people (29). A PPA fall risk is designated mild if the score is between 0 and 1, moderate between 1 and 2, and marked for scores >2. Participants were also asked about any falls experienced in the past 12 months. Falls were defined as unexpected events that resulted in unintentionally coming to the ground, floor, or other lower level (30). A reported history of multiple falls and/or having high physiological fall risk (PPA score ≥1.0) was used to classify participants into the high fall risk group with all other participants classified as low risk. Including past falls in the fall risk classification broadened it beyond physical risk to also encompass behavioral and cognitive facets (31).

### Demographic, Physical, and Clinical Data

Participants completed a questionnaire seeking information on demographics (age, sex, and years of formal education), medical history, and medications. Average amounts of physical activity (both incidental and planned activity including walking) per week over the 3 months prior to the assessment were recorded using the Incidental and Planned Exercise Questionnaire (32). The Addenbrooke's Cognitive Examination-Revised (33) was used to assess global cognition.

### Stepping Tests

**Figure 1:**
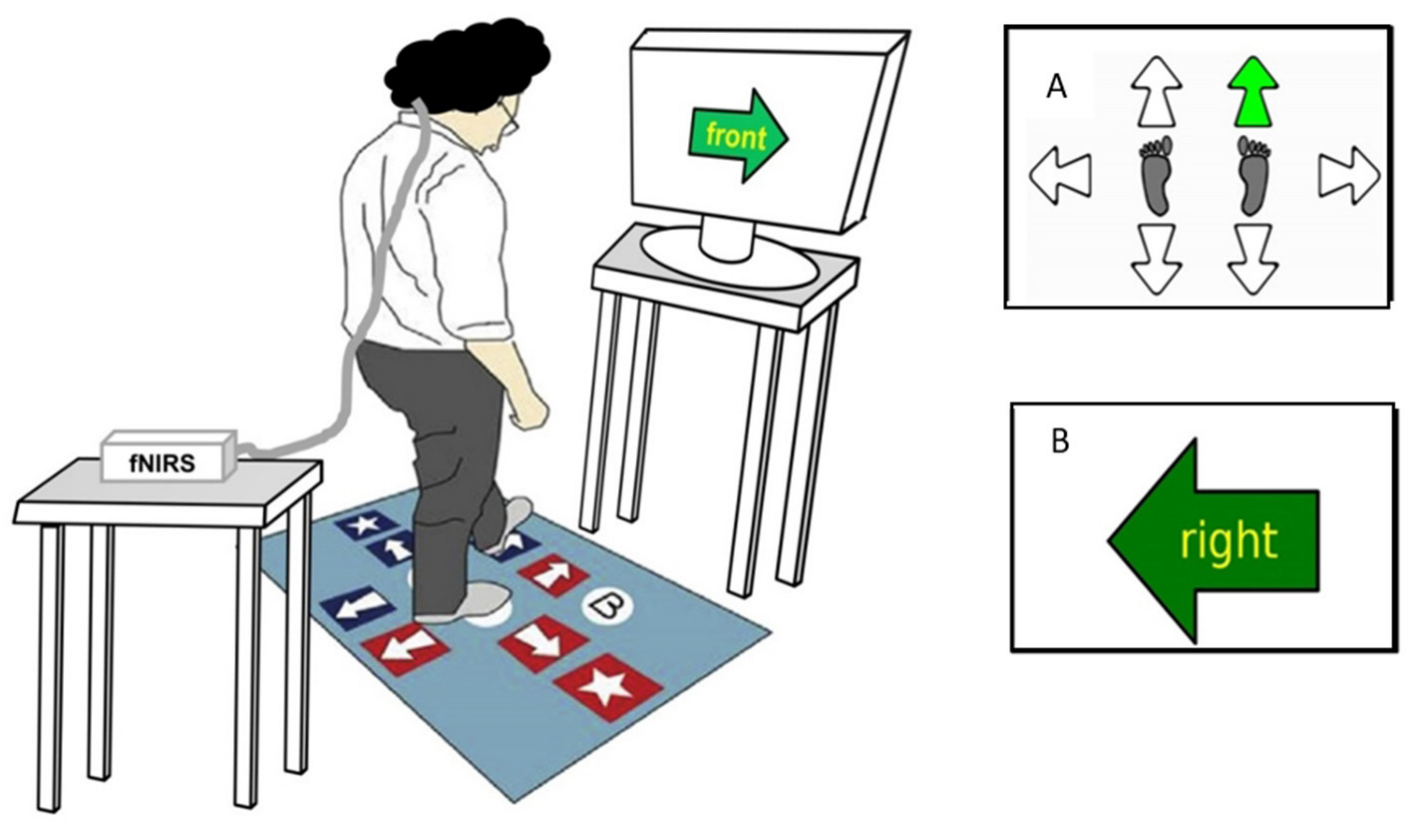
Computerized stepping mat setup and stepping tests display. This figure represents a participant performing the Stroop Stepping Test (SST). The participant stands on the step mat while wearing the fNIRS system on their head with an opaque black cap covering the optodes in order to eliminate the influence of external lights on cortical activity and looks at the monitor screen 1 m ahead. Test conditions include the following: **(A)** Choice-Stepping Reaction Time (CSRT) test, during which participants are required to step as quickly as possible onto the stepping mat panels corresponding to the location of the green arrow appearing (here, right/forward arrow on the mat); and **(B)** SST, during which participants are required to step as quickly as possible on the panel corresponding to the direction defined by the word in the arrow and not the orientation of the arrow itself (here, step on the right panel).

Two stepping tests (CSRT and SST) were conducted using a customized system comprising a computerized mat (150 × 90 cm) and a computer screen (34). The mat contained eight panels: two central stance panels, a left panel, a right panel, two front panels, and two back panels (Figure 1).

For the CSRT test, participants were asked to stand on the two central panels. They were then instructed to step onto a panel as quickly as possible when the corresponding arrow on the screen changed color from white to green (Figure 1A). Participants first undertook 6 practice trials followed by 24 randomly presented trials [4 trials for each of the 6 stepping panels (left, right, front left, front right, back left, and back right)]. An error was defined as a step onto an incorrect panel.

In the SST, a large arrow was presented in the center of the screen pointing in one of four directions (up, down, left, and right) that matched the four possible step directions (forward, backward, left, and right). A word indicating a different direction was written inside the arrow. Participants were instructed to “step by the word” and therefore had to inhibit the response indicated by the arrow's orientation. Four practice trials followed by 20 randomly presented trials (5 trials for each of the 4 corresponding directions) were administered. Errors comprised any steps taken that were not by the word.

Stepping performance measures included the following: mean and intra-individual variability (standard deviation) of response and movement times computed across 24 trials in the CSRT and 20 trials in the SST. Response time was defined as the time from stimulus onset to foot lift-off. Movement time was defined as the time between foot lift-off and touchdown on the correct step panel. The order of the step tests (CSRT and SST) was randomized and all assessments were conducted within one 2-h session.

### fNIRS Data Acquisition and Analysis

Cortical activity while participants performed the stepping tasks was recorded by a continuous-wave fNIRS system (NIRSport, NIRx, Los Angeles, USA). This wearable device contains eight LED sources that emit 760-nm and 850-nm frequency-modulated wavelengths and eight detectors. The sampling rate was set at 7.81 Hz. The 16 optodes, making up 16 channels, were placed on a lightweight cap based on the 10-10 international system. The fNIRS Optodes' Location Decider toolbox (35) and the Brodmann area atlas (36) were used to define the following regions of interest: DLPFC (Brodmann area 9), SMA (Brodmann area 8 or Frontal Eye Fields, which is also covered by part of the SMA), and PMC (Brodmann area 6). Due to a limited number of optodes, we were only able to cover part of these cortical areas. Optode positions, associated channels, anatomic landmarks, and their specificity are outlined in Table 1. We considered coverage of ≥50% of a region of interest sufficient (28). Caps (size 54, 56, or 58 cm) were positioned on the participants' heads, and the Cz position was considered as the reference, centered between the nasion and the inion (anteroposterior measurement) and between the left and right preauricular points (mediolateral measurement). The optodes were covered by an opaque black cap to reduce the interference of external lights.

**Table 1:** Description of the 16 channels of the fNIRS system; source/detector combinations, anatomic locations, and individual channel specificity.

**Channels**	**Source, detector**	**Optodes**	**Brodmann area**	**Anatomic brain region**	**Specificity level (%)**
1	1,1	Fz/Afz	9	DLPFC	61.77
2	1,2	Fz/F1	9	DLPFC	63.16
3	2,2	F3/F1	9	DLPFC	66.61
4	3,2	FC1/F1	8	SMA	63.72
5	1,5	Fz/FCz	8	SMA	60.02
6	3,4	FC1/C1	6	PMC	81.78
7	5,5	Cz/FCz	6	PMC	83.77
8	5,4	Cz/C1	6	PMC	56.45
9	4,3	C3/FC3	6	PMC	61.71
10	1,6	Fz/F2	9	DLPFC	68.93
11	7,6	F4/F2	9	DLPFC	68.37
12	6,6	FC2/F2	8	SMA	58.07
13	6,5	FC2/FCz	6	PMC	62.95
14	6,8	FC2/C2	6	PMC	82.46
15	5,8	Cz/C2	6	PMC	55.39
16	8,7	C4/FC4	6	PMC	56.87

*DLPFC, dorsolateral prefrontal cortex; SMA, supplementary motor area; PMC, premotor cortex*.

The data were recorded using NIRStar 15-2 software. Prior to each trial, the equipment was calibrated to determine the optimal amplification factor to be achieved within an optimal range (0.4–7.0 V). During the calibration, the participants were asked to stand still looking at the computer screen positioned 1 m ahead. The quality of the signals was then evaluated by the amplification gain and signal level. The differential path length factor was adjusted according to each participant's age (37). The experiment started immediately following calibration. fNIRS data acquisition for each of the two stepping tests started with the collection of 30 s of baseline data during which participants were required to stand still (to bring the hemodynamic status as close to a resting state as possible). In sequence after an examiner's verbal command, the participant performed one of the two randomly presented stepping tests.

**Figure 2:**
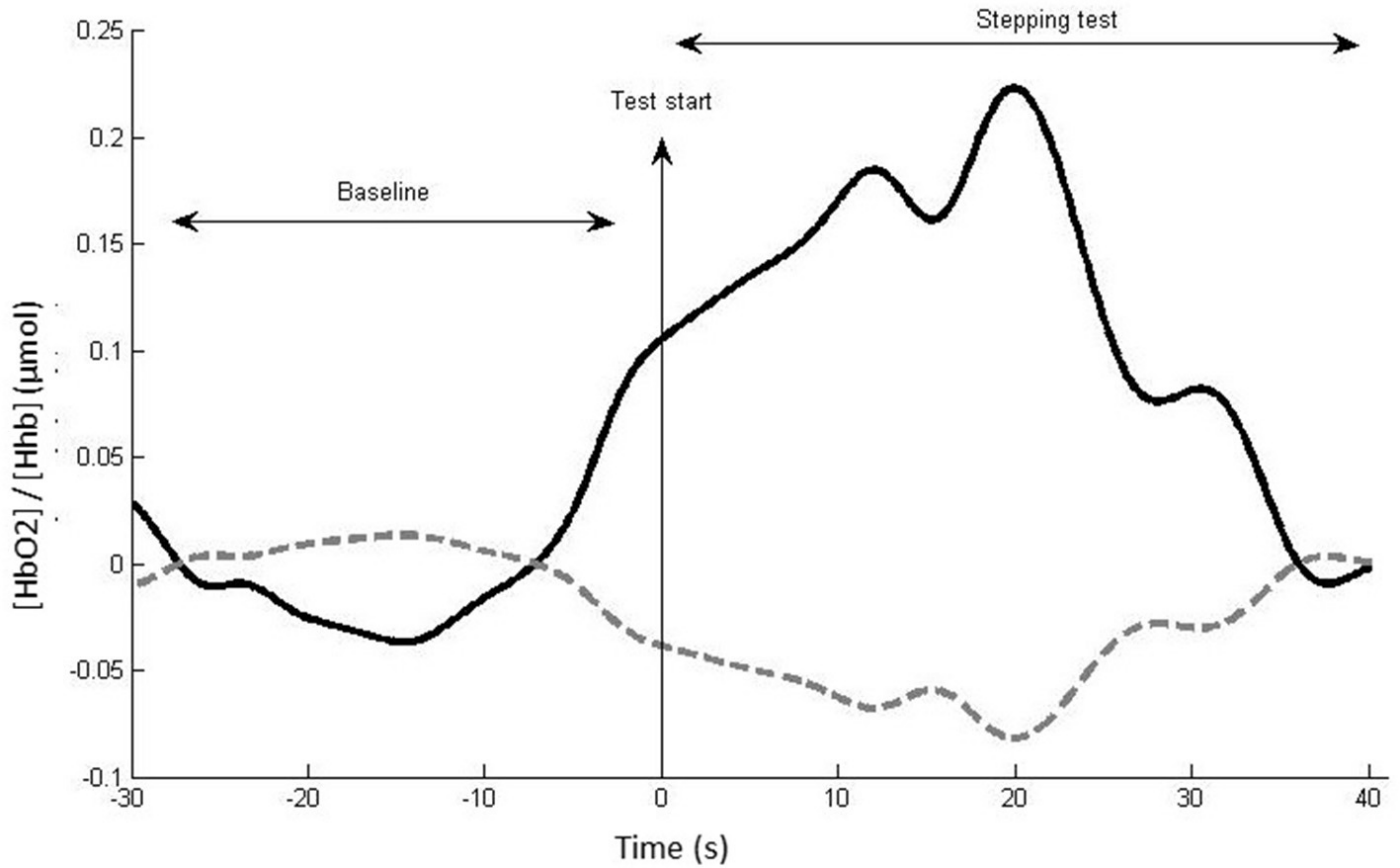
Example of hemodynamic response, with (HbO_2_) (thick black line) and HHb (dotted gray line) for a single channel, participant, and task. The mean (HbO_2_)/(HHb) change between the baseline and test period reflects the neural activity induced by the test condition in this channel.

The fNIRS data were analyzed using Homer2 open-source software in Matlab. The following steps were performed following a recently published guideline (38): (i) raw data were converted to optical density data; (ii) the software excluded the channel as an active channel if the luminous signal was too weak (<0.01 cd) or too strong (>300 cd), if mean data divided by its standard deviation <2, or if the source–detector separation was <0 mm or >45 mm, then; (iii) motion artifacts defined as signal changes greater than a set parameter (standard deviation threshold = 10; amplitude threshold = 0.3) were removed; (iv) wavelet transformation of the optical density data was performed to identify motion artifacts (14, 39, 40) (interquartile range = 0.1); (v) data were filtered with a high-pass filter at 0.01 Hz and a low-pass filter at 0.14 Hz (to remove physiological events, i.e., heart rate) (14, 38); (vi) the optical density data were converted to HbO_2_, HHb, and total concentrations; (vii) a correlation-based signal improvement of the hemoglobin concentration changes was performed to correct for motion artifacts (41); (viii) the length of each stepping test data collection period was standardized for each individual to their shortest stepping test duration (limited up to 60 s); (ix) block averages were computed for HbO_2_, HHb, and total concentrations for each participant, condition, and channel from 30 s preceding the start of the test (baseline period) to the standardized stepping test duration for each participant (maximum 60 s). The difference between the mean HbO_2_, HHb, and total concentration during the baseline period and test period was calculated to obtain relative HbO_2_, HHb, and total concentration values (Figure 2). For each participant and stepping condition, relative HbO_2_, HHb, and total concentrations for the DLPFC, SMA, and PMC were computed as averages across the relevant channels as indicated in Table 1.

### Statistical Analysis

Continuous data were inspected for right skewed distributions, and then log transformed if required, thereby allowing parametric analyses. Outliers were replaced with mean +/− 3SD (one very slow decision time, four very high step time variability, and seven very high HbO2 concentration measures). Student's *t*-tests and chi-square tests were used to assess between-group differences in the demographic, falls, and stepping error data, as well as to compare differences in cortical activity in participants when categorized into groups based on median splits for total stepping reaction time (sum of response and movement times) for the CSRT (median: 1,060 ms) and SST (median: 1,395 ms). Two-way analysis of variance tests were performed for the step performance and hemodynamic data with stepping condition (CSRT vs. SST) as the within-subject factor and group (low fall risk vs. high fall risk) as the between-subject factor. Significance levels were set at 0.05 and statistical trends for the interactions at 0.05 < *p* < 0.1. The data were analyzed using SPSS v. 25 for Windows (SPSS, Inc., Chicago, IL).

## Results

### Demographic, Physical Performance, and Clinical Data

Thirty-nine participants (41%) reported at least one fall in the past 12 months with 14 (15%) reporting two or more falls. Fall risk scores ranged from −4.65 to 2.54 (mean = −0.08; SD = 1.15). Twenty-four participants were classified as having high fall risk (14 with PPA scores ≥ 1.0; 14 with multiple previous falls; 4 with both) and 71 as having low fall risk. There were no between-group differences for sex, age, educational level, or physical activity (*p* > 0.05) (Table 2).

**Table 2:** Demographic and clinical measures. Data are mean (SD) unless stated otherwise.

	**Low fall risk**	**High fall risk**	***p***
	**(*N* = 71)**	**(*N* = 24)**	
Sex (% Men)	17 (24)	6 (25)	0.917
Age (years)	70.9 (5.0)	72.9 (4.5)	0.830
Educational level (years)	15.9 (3.9)	16.7 (5.1)	0.425
Physical activity (hours/week)^a^	32.4 (16.4)	27.8 (16.6)	0.238
Medication intake (*n*)	3.6 (3.0)	4.2 (3.7)	0.415
Number of comorbidities (*n*)	**1.9 (1.8)**	**2.8 (1.9)**	**0.033**
Addenbrooke's Cognitive Examination-Revised (score)	95.7 (3.6)	94.6 (4.1)	0.217

a *Measured with the Incidental and Planned Exercise Questionnaire*.

*Lower scores indicate worse performance in the Incidental and Planned Exercise Questionnaire and in the Addenbrooke's Cognitive Examination-Revised*.

### Stepping Responses

Table 3 presents the mean and intra-individual variability values for the stepping response and movement times for the CSRT and SST. No group × condition interactions or group main effects for mean response or movement times were observed but significant condition main effects indicated that both response and movement times were longer in the SST condition compared to the CSRT test.

**Table 3:** Stepping performance results by group for the choice stepping reaction time (CSRT) test and Stroop stepping test (SST).

		**Low fall risk (*****N*** **=** **71)**		**High fall risk (*****N*** **=** **24)**		**Group main effect, *p*-value**	**Condition main effect, *p*-value**	**Interaction, *p*-value**
		**CSRT**	**SST**	**CSRT**	**SST**			
Response time	Mean	763 (92)	1,072 (220)	784 (73)	1,153 (167)	0.071	**<0.001**	0.101
	IIV	104 (34)	180 (101)	103 (20)	265 (170)	**0.033**	**<0.001**	**0.024**^**a**^
Movement time	Mean	276 (60)	334 (92)	299 (84)	353 (109)	0.120	**<0.001**	0.796
	IIV	78 (41)	181 (158)	81 (37)	224 (191)	0.454	**<0.001**	0.645

*Data are mean (SD)*.

*IIV: intra-individual variability*.

a *Group × Condition interaction, post-hoc test results: SST > CSRT for low fall risk group (p < 0.001); SST > CSRT for high fall risk group (p < 0.001); high fall risk group >low fall risk group for SST (p = 0.012)*.

*Bold values indicate p < 0.05*.

A significant group × condition interaction was observed for intra-individual response time variability (*p* = 0.024) and *post-hoc* analyses indicated the following: intra-individual response time variability was disproportionately higher in the SST condition compared to the CSRT condition in the high fall risk group (*p* = 0.012), and both groups exhibited higher intra-individual response time variability in the SST condition compared to the CSRT condition (*p* < 0.001 for both groups).

Table 4 presents the number of participants who made stepping errors in the two tests. More participants made errors in the SST compared with the CSRT condition (28 participants vs. 6 participants), but there were no between-group differences in either the CSRT (χ^2^ = 0.251, df = 1, *p* = 0.617) or the SST (χ^2^ = 0.001, df = 1, *p* = 0.970).

**Table 4:** Number (%) of participants who made at least one stepping error in the choice stepping reaction time (CSRT) test and Stroop stepping test (SST).

	**Low fall risk**	**High fall risk**	*p*-value^*^
	**(*N* = 71)**	**(*N* = 24)**	
CSRT	5 (7.0)	1 (4.2)	0.617
SST	21 (29.6)	7 (29.2)	0.970

* *From Chi-square tests*.

### Cortical Activity

For simplicity, only the HbO_2_ concentration data are presented in the main body of this paper (Table 5), with the data for HHb and total hemoglobin concentrations presented as Supplementary Table 1. A group × condition interaction trend (0.05 < *p* < 0.1) was observed for mean HbO_2_ concentration in the DLPFC (*p* = 0.095) and *post-hoc* analysis revealed that the high fall risk group had greater HbO_2_ concentration increases in the SST condition compared to the low fall risk group (*p* = 0.047) and compared with the CSRT condition (*p* = 0.002). Additional main effects revealed that both groups had higher SMA and PMC cortical activity in the SST condition compared to the CSRT condition. The HHb and total hemoglobin concentration findings were generally consistent with the HbO_2_ findings, with a statistically significant *post-hoc* test for the group × condition interaction regarding HHb concentration in the DLPFC and indicating significantly greater activity in the DLPFC (reduced HHb concentration) for the high fall risk group during the SST compared with the CSRT (Supplementary Table 1).

**Table 5:** Relative oxyhemoglobin (HbO2) concentration (μmol/L) in the cortical regions of interest by group in the choice stepping reaction time (CSRT) test and Stroop stepping test (SST).

	**Low fall risk (*****N*** **=** **71)**		**High fall risk (*****N*** **=** **24)**		**Group main effect**	**Condition main effect**	**Interaction**
	**CSRT**	**SST**	**CSRT**	**SST**			
DLPFC	0.019 (0.041)	0.031 (0.042)	0.023 (0.063)	0.055 (0.065)	0.176	**0.001**	*0.095*^**a**^
SMA	0.023 (0.034)	0.038 (0.047)	0.033 (0.062)	0.051 (0.076)	0.269	**0.006**	0.869
PMC	0.034 (0.044)	0.053 (0.053)	0.036 (0.060)	0.062 (0.070)	0.608	**0.001**	0.588

*Data are mean (SD)*.

*DLPFC, dorsolateral prefrontal cortex; SMA, supplementary motor area; PMC, premotor cortex; HbO2, oxyhemoglobin concentration*.

a *Group × Condition interaction, post-hoc test results: SST > CSRT for low fall risk group (p = 0.039); SST > CSRT for high fall risk group (p = 0.002); high fall risk > low fall risk for SST (p = 0.047)*.

*Bold values indicate p < 0.05*.

*Italic values indicate 0.05 < p < 0.1*.

Additional comparisons of cortical activity between slow and fast CSRT and SST performers showed no between-group activity differences in the DLPFC, SMA, and PMC (Supplementary Table 2).

## Discussion

CSRT tasks are underpinned by neuropsychological, sensorimotor, and balance systems and therefore offer good indices of fall risk and physical and cognitive frailty (7, 19, 34, 42). In this study, we found that compared to their low-risk counterparts, older people at high fall risk had greater DLPFC activity and increased intra-individual stepping response times during the performance of SST. Furthermore, significant main effects of condition confirmed that the SST performance generated slower mean response times and slower and more variable movement times and required increased recruitment of the SMA and PMC, compared with the CSRT. These findings are in line with but only partly confirm our *a priori* hypotheses as discussed below.

All participants had slower and more variable response times when performing the SST compared to the CSRT task, likely due to the SST incorporating conflict resolution and inhibition in addition to attention. Previously, we reported that slower SST times were significantly correlated with poorer executive function as assessed with the Digit Symbol Substitution Test, Trail-making test, and the Victoria Stroop test (7). Our current findings showing significant increases in relative HbO_2_ concentration in three brain regions of interest suggest that performing the SST requires increased activation across the indirect locomotor pathway, that is, attention and executive functioning (DLPFC) (14, 20), executive planning and generation of anticipatory postural adjustments (SMA) (21–23), and motor sequencing in response to external stimuli (PMC) (24). This pattern of concurrent hemodynamic response augmentation in the DLPFC, SMA, and PMC builds on the findings of previous studies that have contrasted walking tasks that differ with respect to cognitive-motor complexity, showing increased cortical activity with greater task complexity (12–14).

As hypothesized, the high fall risk group had more variable stepping response times than the low fall risk group evident in the more complex SST. This finding is consistent with our past studies involving older people that show that both the CSRT and SST significantly discriminate between fallers and non-fallers (7, 19) and that high intra-individual variability in stepping responses is associated with falls in older people with mild cognitive impairment (35). Our concomitant hemodynamic results indicating that the high fall risk group exhibited higher DLPFC activity in the SST task align with the neural inefficiency model that proposes cortical over-activation occurs in parallel with reduced task performance (25, 43, 44). Therefore, it is possible that reduced brain structural integrity and functional connectivity (“less wiring, more firing”) (44) results in inefficient activation of cortical circuits and contributes to the poorer stepping performance in our high-risk faller group.

Our hemodynamic findings may also reflect a compensatory process to overcome sensorimotor impairments and/or declining brain capacity (25, 26, 45, 46), as well as the need to allocate more attentional resources to deal with task complexity (47). A previous stepping study using fNIRS in young adults demonstrated that a choice reaction time task elicited greater DLPFC activity than a simple reaction time task (17). Our results are in line with those of Verghese et al. (15) who found that increased DLPFC activity when performing a cognitively complex task while walking predicted falls in older adults. Similar patterns of compensatory increased SMA activation have been noted in older people vs. younger people, when undertaking imagined walking during functional magnetic resonance imaging (48). It has also been reported that the SMA plays a role in ankle joint motor preparation required for stepping responses to visual stimuli (49) and the generation of anticipatory postural adjustments (16, 22, 23)—both relevant functions to the execution of volitional stepping as required in the CSRT and SST tasks. In a previous study that investigated cortical activity concurrently with balance perturbations, increased SMA activity was correlated with reduced ML sway in young adults required to keep maintain their balance on a balance board, supporting the role of the SMA in the online control of ML sway (50). Finally, there is evidence of involvement of the PMC in volitional tasks that require postural control (51) and in the control of locomotion (52) and that greater amyloid deposition in the PMC is associated with increased gait variability (53).

High between-subject variability, inherent to fNIRS data (54), might have attenuated significant effects that would only become apparent with greater statistical power. We also acknowledge other study limitations. First, we used the fNIRS Optodes' Location Decider toolbox based on the Brodmann atlas classification (35) to identify the anatomical landmarks for optode placement. More recent recommended techniques based on MRI mapping may have improved the region of interest placement precision. Second, the use of data-based filters at the processing stage as opposed to short-separation channels (which measure the extracerebral activity alone, so that it may be removed from the total fNIRS signal) means that we cannot guarantee the complete removal of physiological and motion artifacts. Given that we cannot rule out superficial blood flow contamination, our findings should be interpreted with due caution. Finally, the categorization of fall risk was based on falls experienced within the past year and physical tests. It is therefore necessary to examine the validity of the findings by investigating neural correlates of stepping tasks in relation to prospectively measured falls.

With respect to clinical application, our finding of increased activity in the DLPFC during the performance of a cognitively demanding stepping task has implications for strategies aimed at improving balance and reducing fall risk in older adults. Specific interventions focusing on improving volitional stepping responses, such as cognitive-motor step training (27), may improve older people's balance through (i) faster and more efficient volitional stepping responses (reduced variability); (ii) improved control of inhibitory responses, step planning and initiation, and in consequence increased efficiency of the DLPFC, SMA and PMC; and (iii) reduced reliance on “cognitive reserve” (47).

In conclusion, older people at high fall risk exhibited increased DLPFC activity and response time variability when completing a cognitively demanding stepping test compared to those at low risk of falls. This increased hemodynamic response might comprise a compensatory process for postural control deficits and/or reflect a degree of neural inefficiency in the DLPFC. Interventions focused on the training of cognitively demanding stepping tasks to enhance motor and neural efficiency and balance should be investigated in the context of frailty and fall prevention.

## Data Availability Statement

The raw data supporting the conclusions of this article will be made available by the authors, without undue reservation.

## Ethics Statement

The studies involving human participants were reviewed and approved by University of New South Wales Human Research Ethics Committee. The patients/participants provided their written informed consent to participate in this study.

## Author Contributions

PP, SL, and JM conceived the study objectives and designed the study. DS provided access to the participants. PP, BH, and JM acquired the data. PP and JM analyzed the data. PP, SL, and JM interpreted the data. PP, SL and JM drafted the manuscript. All authors were involved with subsequent edits and revisions of the manuscript.

## Conflict of Interest

The authors declare that the research was conducted in the absence of any commercial or financial relationships that could be construed as a potential conflict of interest.
