# Supplementary material for: Cognitive and Motor Cortical Activity During Cognitively Demanding Stepping Tasks in Older People at Low and High Risk of Falling
Source: Front Med (Lausanne). 2021 Jul 12;8:554231. doi: 10.3389/fmed.2021.554231 (PMC8310929; doi:10.3389/fmed.2021.554231)
Supplement: Supplementary file 1 [file Table_1.DOCX]

**Table S1.** Relative deoxy-haemoglobin (HHb) and total haemoglobin concentrations (μmol/l) in the cortical regions of interest by group in the Choice Stepping Reaction Time (CSRT) test and Stroop Stepping Test (SST). Data are mean (SD).

|  |  | **Low fall risk (N=71)** | | **High fall risk (N=24)** | | **Group main effect** | **Condition main effect** | **Interaction** |
| --- | --- | --- | --- | --- | --- | --- | --- | --- |
|  |  | **CSRT** | **SST** | **CSRT** | **SST** |  |  |  |
| **DLPFC** | **HHb** | -0.007 (0.014) | -0.010 (0.013) | -0.005 (0.018) | -0.016 (0.019) | 0.530 | **0.001** | *0.062****^a^*** |
|  | **Total** | 0.013 (0.028) | 0.021 (0.029) | 0.018 (0.047) | 0.039 (0.049) | 0.117 | **<0.001** | 0.124 |
|  |  |  |  |  |  |  |  |  |
| **SMA** | **HHb** | -0.008 (0.011) | -0.014 (0.017) | -0.008 (0.022) | -0.013 (0.027) | 0.801 | **0.014** | 0.738 |
|  | **Total** | 0.015 (0.024) | 0.024 (0.032) | 0.025 (0.042) | 0.038 (0.053) | 0.080 | **0.004** | 0.617 |
|  |  |  |  |  |  |  |  |  |
| **PMC** | **HHb** | -0.014 (0.019) | -0.022 (0.024) | -0.011 (0.020) | -0.021 (0.028) | 0.605 | **0.001** | 0.551 |
|  | **Total** | 0.019 (0.026) | 0.031 (0.034) | 0.025 (0.041) | 0.041 (0.045) | 0.231 | **0.001** | 0.685 |

DLPFC: dorsolateral prefrontal cortex; SMA: supplementary motor area; PMC: premotor cortex; HHb: deoxy-haemoglobin concentration.

^a^ Group x Condition interaction: SST > CSRT for high fall risk group (p=0.002);
