# Supplementary material for: Cognitive and Motor Cortical Activity During Cognitively Demanding Stepping Tasks in Older People at Low and High Risk of Falling
Source: Front Med (Lausanne). 2021 Jul 12;8:554231. doi: 10.3389/fmed.2021.554231 (PMC8310929; doi:10.3389/fmed.2021.554231)
Supplement: Supplementary file 2 [file Table_2.DOCX]

**Table S2.** HbO2 concentrations (μmol/l) for slow and faster CSRT and SST performers. Data are mean (SD).

|  | **Slow CSRT (n=48)** | **Fast CSRT (n=47)** | **p** | **Slow SST (n=48)** | **Fast SST (n=47)** | **p** |
| --- | --- | --- | --- | --- | --- | --- |
| **DLPFC** | 0.015 (0.051) | 0.026 (0.043) | 0.271 | 0.039 (0.058) | 0.036 (0.040) | 0.764 |
| **SMA** | 0.027 (0.054) | 0.024 (0.026) | 0.723 | 0.047 (0.068) | 0.036 (0.041) | 0.308 |
| **PMC** | 0.032 (0.051) | 0.037 (0.046) | 0.678 | 0.055 (0.066) | 0.056 (0.049) | 0.936 |

DLPFC: dorsolateral prefrontal cortex; SMA: supplementary motor area; PMC: premotor cortex; CSRT: choice-stepping reaction time test; SST: stroop stepping test.

No between-group differences were observed for slow and faster CSRT and SST performers in the three regions of interest when undertaking the CSRT test and the SST, respectively. Although poor stepping responses are likely associated with neural impairments, our null finding may have been due to the simple categorisation of participants into two groups based on median splits and not taking into account other task-related factors such as errors made in the execution of the stepping tasks. Future studies could investigate associations between cortical activity during stepping task responses and physiological and neuropsychological factors to elucidate such mechanisms.
